# Clinical Case Studies in Psychoanalytic and Psychodynamic Treatment

**DOI:** 10.3389/fpsyg.2017.00108

**Published:** 2017-02-02

**Authors:** Jochem Willemsen, Elena Della Rosa, Sue Kegerreis

**Affiliations:** Centre for Psychoanalytic Studies, University of EssexColchester, UK

**Keywords:** clinical case study, methodology, psychotherapy research, psychoanalysis, psychoanalytic schools, theoretical pluralism, review

## Abstract

This manuscript provides a review of the clinical case study within the field of psychoanalytic and psychodynamic treatment. The method has been contested for methodological reasons and because it would contribute to theoretical pluralism in the field. We summarize how the case study method is being applied in different schools of psychoanalysis, and we clarify the unique strengths of this method and areas for improvement. Finally, based on the literature and on our own experience with case study research, we come to formulate nine guidelines for future case study authors: (1) basic information to include, (2) clarification of the motivation to select a particular patient, (3) information about informed consent and disguise, (4) patient background and context of referral or self-referral, (5) patient's narrative, therapist's observations and interpretations, (6) interpretative heuristics, (7) reflexivity and counter-transference, (8) leaving room for interpretation, and (9) answering the research question, and comparison with other cases.

## Introduction

Psychoanalysis has always been, according to its inventor, both a research endeavor and a therapeutic endeavor. Furthermore it is clear from Freud's autobiography that he prioritized the research aspect; he did not become a doctor because he wished to cure people in ill health (Freud, 2001 [1925]). His invention of the psychoanalytic approach to therapy, involving the patient lying down and associating freely, served a research purpose as much as a therapeutic purpose. Through free association, he would be able to gain unique insight in the human mind. Next, he had to find a format to report on his findings, and this would be the case study. The case study method already existed in medicine (Forrester, 2016), but Freud adjusted it considerably. Case studies in medical settings were more like case files, in which the patient was described or reduced to a number of medical categories: the patient became a case of some particular ailment (Forrester, 2016). In Freud's hands, the case study developed into *Kranken Geschichten* in which the current pathology of the patient is related to the whole of his life, sometimes even over generations.

Although Freud's case studies have demonstrably provided data for generations of research by analysts (Midgley, 2006a) and various scholars (Pletsch, 1982; Sealey, 2011; Damousi et al., 2015), the method of the case study has become very controversial. According to Midgley (2006b), objections against the case study method can be grouped into three arguments. First there is the data problem: case studies provide no objective clinical data (Widlöcher, 1994), they only report on what went right and disregard any confusion or mistakes (Spence, 2001). Second, there is the data analysis problem: the way in which the observations of the case study are analyzed lack validity; case studies confirm what we already know (Spence, 2001). Some go even so far to say that they are purely subjective: Michels calls case studies the “crystallization of the analyst's countertransference” (Michels, 2000, p. 373). Thirdly, there is the generalizability problem: it is not possible to gain generalizable insight from case studies. Reading, writing and presenting case studies has been described as being a group ritual to affirm analysts in their professional identity, rather than a research method (Widlöcher, 1994).

These criticisms stand in contrast to the respect gained by the case study method in the last two decades. Since the 1990s there has been an increasing number of psychoanalytic and psychodynamic **clinical case study** and **empirical case studies** being published in scientific journals (Desmet et al., 2013; Cornelis et al., in press). It has also been signaled that the case study method is being revived more broadly in the social sciences. In the most recent, fifth edition of his seminal book on case study research, Yinn (2014) includes a figure showing the steady increase of the frequency with which the term “case study research” appears in published books in the period from 1980 to 2008.

KEY CONCEPT 1 **Clinical case study**

A clinical case study is a narrative report by the therapist of what happened during a therapy together with the therapist's interpretations of what happened. It is possible that certain (semi)-structured assessment instruments, such as a questionnaire or a diagnostic interview are included in clinical case studies, yet it is still the therapist that uses these, interprets and discusses them.

KEY CONCEPT 2 **Empirical case studies**

In an empirical case study data are gathered from different sources (e.g., self-report, observation,…) and there is a research team involved in the analyses of the data. This study can take place either in a naturalistic setting (systematic case study) or in a controlled experimental environment (single-case experiment).

In addition to the controversy about the case study method, psychoanalysis has developed into a fragmented discipline. The different psychoanalytic schools share Freud's idea of the unconscious mind, but they focus on different aspects in his theoretical work. Some of the schools still operate under the wings of the International Psychoanalytic Association, while others have established their own global association. Each school is linked to one or several key psychoanalysts who have developed their own version of psychoanalysis. Each psychoanalytic school has a different set of theories but there are also differences in the training of new psychoanalysts and in the therapeutic techniques that are applied by its proponents.

Based on this heterogeneity of perspectives in psychoanalysis, a research group around the **Single Case Archive** investigated the current status of case study research in psychoanalysis (Willemsen et al., 2015a). They were particularly interested to know more about the output and methodology of case studies within the different psychoanalytic schools.

KEY CONCEPT 3 **Single case archive**

The Single Case Archive is an online archive of published clinical and empirical case studies in the field of psychotherapy (http://www.singlecasearchive.com). The objective of this archive is to facilitate the study of case studies for research, clinical, and teaching purposes. The online search engine allows the identification of sets of cases in function of specific clinical or research questions.

## Our survey among case study authors about their psychoanalytic school

In order to investigate and compare case studies from different psychoanalytic schools, we first had to find a way of identifying to which school the case studies belonged. This is very difficult to judge straightforwardly on the basis of the published case study: the fact that someone cites Winnicott or makes transference interpretations doesn't place him or her firmly within a particular psychoanalytic school. The best approach was to ask the authors themselves. Therefore, we contacted all case study authors included in the Single Case Archive (since the time of our original study in 2013, the archive has expanded). We sent emails and letters in different languages to 445 authors and received 200 replies (45% response rate). We asked them the following question: “*At the time you were working on this specific case, to which psychoanalytic school(s) did you feel most attached?*” Each author was given 10 options: (1) Self Psychology (1.a Theory of Heinz Kohut, 1.b Post-Kohutian Theories, 1.c Intersubjective psychoanalysis), (2) Relational psychoanalysis, (3) Interpersonal psychoanalysis, (4) Object relational psychoanalysis (4.a Theory of Melanie Klein, 4.b Theory of Donald W. Winnicott, 4.c Theory of Wilfred R. Bion, 4.d Theory of Otto F. Kernberg), (5) Ego psychology (or) “Classic psychoanalysis” (5.a Theories of Sigmund Freud, 5.b Ego psychology, 5.c Post-Ego psychology), (6) Lacanian psychoanalysis, (7) Jungian psychoanalysis, (8) National Psychological Association for Psychoanalysis (NPAP) related theory, (9) Modern psychoanalysis related to the Boston or New York Graduate School of Psychoanalysis (BGSP/NYGSP), (10) Other. Respondents could indicate one or more options.

Analysis of the responses indicated that the two oldest schools in psychoanalysis, Object-relations psychoanalysis and Ego psychology, dominate the field in relation to case studies that are published in scientific journals. More than three quarters of all case study authors (77%) reported these schools of thought to be the ones with which they considered themselves most affiliated. Three more recent schools were also well-represented among case studies: Self Psychology, Relational Psychoanalysis, and Interpersonal Psychoanalysis. Lacanian Psychoanalysis, Jungian Psychoanalysis, NPAP related Theory and Modern Psychoanalysis related to the BGSP/NYGSP were only rarely mentioned by case study authors as their school of thought. This does not mean that clinicians or researchers within these latter schools do not write any case studies. It only means that they publish few case studies in the scientific journals included in ISI-ranked journals indexed in Web of Science. But they might have their own journals in which they publish clinical material.

Our survey demonstrated that the majority of case study authors (59%) feel attached to more than one psychoanalytic school. This was in fact one of the surprising findings in our study. It seems that **theoretical pluralism** is more rule than exception among case study authors. There were some differences between the psychoanalytic schools though in terms of pluralism. Case study authors who feel attached to Self Psychology and Interpersonal Psychoanalysis are the most pluralistic: 92 and 86%, respectively also affiliate with one or more other psychoanalytic schools. Case study authors who feel attached to Object Relations Psychoanalysis are the “purest” group: only 69% of them affiliate with one or more other psychoanalytic schools.

KEY CONCEPT 4 **Theoretical pluralism**

A situation in which several, potentially contradicting, theories coexist. It is sometimes interpreted as a sign of the immaturity of a science, under the assumption that a mature science should arrive at one single coherent truth. Others see theoretical pluralism as unavoidable for any applied discipline, as each theory can highlight only part of reality.

## Psychoanalytic pluralism and the case study method

We were not really surprised to find that Object Relations psychoanalysis and Ego psychology were the most dominant schools in the field of psychoanalytic case studies, as they are very present in European, Latin-American and North-American psychoanalytic institutes. We were more surprised to find such a high degree of pluralism among these case study authors, given the fact that disputes between analysts from different schools can be quite ardent (Green, 2005; Summers, 2008). Others have compared the situation of psychoanalytic schools with the Tower of Babel (Steiner, 1994).

It has been argued that the case study method contributes to the degree of theoretical pluralism within psychoanalysis. The reason for this is situated in the reasoning style at the basis of case study research (Chiesa, 2010; Fonagy, 2015). The author of a psychoanalytic case study makes a number of observations about the patient within the context of the treatment, and then moves to a conclusion about the patient's psychodynamics in general. The conclusion he or she arrives at inductively gains its “truth value” from the number and quality of observations it is based on. This style of reasoning in case study research is very similar to how clinicians reason in general. Clinicians look for patterns within patients and across patients. If they make similar observations in different patients, or if other psychoanalysts make similar observations in their patients, the weight of the conclusion becomes greater and greater. The problem with this reasoning style is that one can never arrive at definite conclusions: even if a conclusion is based on a large number of observations, it is always possible that the next observation disconfirms the conclusion. Therefore, it could be said, it is impossible to attain “true” knowledge.

The above argument is basically similar to objections against any kind of qualitative research. To this, we argue with Rustin (2003) that there is not one science and no hierarchy of research methods. Each method comes with strengths and weaknesses, and what one gains in terms of control and certainty in a conventional experimental setup is lost in terms of external validity and clinical applicability. Numerous researchers have pleaded for the case study approach as one method among a whole range of research methods in the field of psychoanalysis (Rustin, 2003; Luyten et al., 2006; Midgley, 2006b; Colombo and Michels, 2007; Vanheule, 2009; Hinshelwood, 2013). Leuzinger-Bohleber makes a distinction between clinical research and extra-clinical research (Leuzinger-Bohleber, 2015). Clinical research is the idiographic type of research conducted by a psychoanalyst who is working with a patient. Unconscious phantasies and conflicts are symbolized and put into words at different levels of abstraction. This understanding then molds the perception of the analyst in subsequent clinical situations; even though the basic psychoanalytic attitude of “not knowing” is maintained. The clinical case study is clinical research *par excellence*. Extra-clinical research consists in the application of different methodologies developed in the natural and human sciences, to the study of the unconscious mind. Leuzinger-Bohleber refers to empirical psychotherapy research, experimental research, literature, cultural studies, etc. We believe that the clinical case study method should step up and claim its place in psychoanalytic research, although we agree that the method should be developed further. This paper and a number of others such as Midgley (2006b) should facilitate this methodological improvement. The clinical research method is very well-suited to address any research question related to the description of phenomena and sequences in psychotherapy (e.g., manifestation and evolution of symptoms and therapeutic relationship over time). It is not suitable for questions related to causality and outcome.

We also want to point out that there is a new evolution in the field of psychotherapy case study research, which consists in the development of methodologies for **meta-studies of clinical case studies** (Iwakabe and Gazzola, 2009). The evolution builds on the broader tendency in the field of qualitative research to work toward integration or synthesis of qualitative findings (Finfgeld, 2003; Zimmer, 2006). The first studies which use this methodology have been published recently: Widdowson (2016) developed a treatment manual for depression, Rabinovich (2016) studied the integration of behavioral and psychoanalytic treatment interventions, and Willemsen et al. (2015b) investigated patterns of transference in perversion. The rich variety of research aims demonstrates the potential of these meta-studies of case studies.

KEY CONCEPT 5 **Meta-studies of clinical case studies**

A meta-study of clinical case studies is a research approach in which findings from cases are aggregated and more general patterns in psychotherapeutic processes are described. Several methodologies for meta-studies have been described, including cross-case analysis of raw data, meta-analysis, meta-synthesis, case comparisons, and review studies in general.

## Lack of basic information in psychoanalytic case studies

The second research question of our study (Willemsen et al., 2015a) concerned the methodological, patient, therapist, and treatment characteristics of published psychoanalytic case studies. All studies included in the Single Case Archive are screened by means of a coding sheet for basic information, the Inventory of Basic Information in Single Cases (IBISC). The IBISC was designed to assess the presence of basic information on patient (e.g., age, gender, reasons to consult), therapist (e.g., age, gender, level of experience), treatment (e.g., duration, frequency, outcome), and the methodology (e.g., therapy notes or audio recoding of sessions). The IBISC coding revealed that a lot of basic information is simply missing in psychoanalytic case studies (Desmet et al., 2013). Patient information is fairly well-reported, but information about therapist, treatment and methodology are often totally absent. Training and years of experience are not mentioned in 84 and 94% of the cases, respectively. The setting of the treatment is not mentioned in 61% of the case studies. In 80% of the cases, it was not mentioned whether the writing of the case studies was on the basis of therapy notes, or audiotapes. In 91% of the cases, it was not mentioned whether informed consent was obtained.

Using variables on which we had more comprehensive information, we compared basic information of case studies from different psychoanalytic schools. This gave us a more detailed insight in the type of case studies that have been generated within each psychoanalytic school, and into the difference between these schools in terms of the kind of case study they generate. We found only minimal differences. Case studies in Relational Psychoanalysis stand out because they involve older patients and longer treatments. Case studies in Interpersonal Psychoanalysis tend to involve young, female patients and male therapists. Case study authors from both these schools tend to report on intensive psychoanalysis in terms of session frequency. But for the rest, it seems that the publication of case studies throughout the different psychoanalytic schools has intensified quite recently.

## Guidelines for writing clinical case studies

One of the main problems in using psychoanalytic case studies for research purposes is the enormous variability in quality of reporting and inconsistency in the provision of basic information about the case. This prevents the reader from contextualizing the case study and it obstructs the comparison of one case study with another. There have been attempts to provide guidelines for the writing of case studies, especially in the context of analytic training within the American Psychoanalytic Association (Klumpner and Frank, 1991; Bernstein, 2008). However, these guidelines were never enforced for case study authors by the editors from the main psychoanalytic journals. Therefore, the impact of these guidelines on the field of case study research has remained limited.

Here at the end of our focused review, we would like to provide guidelines for future case study authors. Our guidelines are based on the literature and on our experience with reading, writing, and doing research with clinical case studies. We will include fragments of existing case studies to clarify our guidelines. These guidelines do not provide a structure or framework for the case study; they set out basic principles about what should be included in a case study.

### Basic information

First of all, we think that a clinical case study needs to contain basic information about the patient, the therapist, the treatment, and the research method. In relation to the *patient*, it is relevant to report on gender, age (or an age range in which to situate the patient), and ethnicity or cultural background. The reader needs to know these characteristics in order to orientate themselves as to who the patient is and what brings them to therapy. In relation to the *therapist*, it is important to provide information about professional training, level of professional experience, and theoretical orientation. Tuckett (2008) emphasizes the importance for clinicians to be explicit about the theory they are using and about their way of practicing. It is not sufficient to state membership of a particular group or school, because most groups have a wide range of different ways of practicing. In relation to the *treatment* itself, it is important to be explicit about the kind of setting, the duration of treatment, the frequency of sessions, and details about separate sequences in the treatment (diagnostic phase, follow-up etc.). These are essential features to share, especially at a time when public sector mental health treatment is being subjected to tight time restrictions and particular ways of practising are favored over others. For example short-term psychotherapies are being implemented in public services for social and economic reasons. While case studies carried out in the public sector can give us information on those short-term therapies, private practice can offer details about the patient's progress on a long-term basis. Moreover, it is important to report whether the treatment is completed. To our astonishment, there are a considerable number of published case studies on therapies that were not finished (Desmet et al., 2013). As Freud (2001 [1909], p. 132) already advised, it is best to wait till completion of the treatment before one starts to work on a case study. Finally, in relation to the *research method*, it is crucial to mention which type of data were collected (therapy notes taken after each session, audio-recordings, questionnaires, etc.), whether informed consent was given, and in what way the treatment was supervised. Clinicians who would like to have help with checking whether they included all necessary basic information case use the Inventory for Basic Information in Single Cases (IBISC), which is freely available on http://www.singlecasearchive.com/resources.

### Motivation to select a particular patient

First of all, it is crucial to know what the motivation for writing about a particular case comes from. Some of the following questions should be kept in mind and made explicit from the beginning of the case presentation. Why is it interesting to look at this case? What is it about this case or the psychotherapist's work that can contribute to the already existing knowledge or technique?

“This treatment resulted in the amelioration of his [obsessive-compulsive] symptoms, which remained stable eight years after treatment ended. Because the standard of care in such cases has become largely behavioral and pharmacological, I will discuss some questions about our current understanding of obsessive-compulsive phenomena that are raised by this case, and some of the factors that likely contributed to the success of psychoanalytic treatment for this child (McGehee, 2005, p. 213–214).”

This quotation refers to a case that has been selected on the basis of its successful outcome. The author is then interested to find out what made this case successful.

### Informed consent and disguise

As regulations on privacy and ethics are becoming tighter, psychotherapists find themselves with a real problem in deciding what is publishable and what is not. Winship (2007) points out that there is a potential negative effect of research overregulation as clinicians may be discouraged from reporting ordinary and everyday findings from their clinical practice. But he also offers very good guidelines for approaching the issue of informed consent. A good practice is asking for consent either at the start of the treatment or after completion of the treatment: preferably not during treatment. It is inadvisable to complete the case study before the treatment has ended. It is also advisable that the process of negotiating consent with the patient is reported in the case study.

“To be sure that Belle's anonymity was preserved, I contacted her while writing this book and told her it would not be published without her complete approval. To do this, I asked if she would review every word of every draft. She has (Stoller, 1986, p. 217).”

In relation to disguise, one has to strike a balance between thin and thick disguise. Gabbard (2000) suggests different useful approaches to disguising the identity of the patient.

### Patient background and context of referral or self-referral

It is important to include relevant facts about the patient's childhood, family history, siblings, any trauma or losses and relationship history (social and romantic) and the current context of the patient's life (family, working, financial). The context of referral is also key to understanding how and why the patient has come to therapy. Was the patient encouraged to come or had wanted to come? Has there been a recent crisis which prompted the intervention or an on-going problem which the patient had wanted to address for some time?

“Michael was one of the youngest children in his family of origin. He had older brothers and sisters who had been received into care before his birth. His parents separated before he was born. There had been some history of violence between them and Michael was received into care on a place of safety order when he was an infant because his mother had been unable to show consistent care toward him (Lykins Trevatt, 1999, p. 267).”

### Patient's narrative, therapist's observations, and interpretations

A case study should contain detailed accounts of key moments or central topics, such as a literal transcription of an interaction between patient and therapist, the narration of a dream, a detailed account of associations, etc. This will increase the fidelity of the case studied, especially when both patient's and therapist's speech are reported as carefully as possible.

“Martha spoke in a high-pitched voice which sounded even more tense than usual. She explained that her best friend's mum had shouted at her for being so withdrawn; this made her angry and left her feeling that she wanted to leave their home for good. I told Martha that she often tried to undo her bad feelings by acting quickly on her instincts, as she did not feel able to hold her feelings in her mind and bring them to her therapy to think about with me. Martha nodded but it was not clear whether she could really think about what I just said to her. She then said that she was being held in the hospital until a new foster placement could be found. “In the meantime,” she said in a pleased tone, “I have to be under constant supervision” (Della Rosa, 2015, p. 168).”

In this example, observations of nonverbal behavior and tonality are also included, which helps to render a lively picture of the interaction.

### Interpretative heuristics

In which frame of reference is the writer operating? It is important to know what theories are guiding the therapist's thinking and what strategies he employs in order to deal with the clinical situation he is encountering. Tuckett (1993) writes about the importance of knowing what “explanatory model” is used by the therapist in order to make sense of the patient and to relate his own thinking to a wider public for the purpose of research. This idea is also supported by Colombo and Michels (2007) who believe that making theoretical orientations as explicit as possible would make the case studies intelligible and more easily employed by the research community. This can be done by the therapists explaining why they have interpreted a particular situation in the way they have. For example, Kegerreis in her paper on time and lateness (2013) stresses throughout how she is working within the object-relations framework and looking out for the patient's use of projective mechanisms.

“She was 10 minutes late. Smiling rather smugly to herself she told me that the wood supplied for her new floor had been wrongly cut. The suppliers were supposed to come and collect it and hadn't done so, so she had told them she was going to sell it to a friend, and they are now all anxious and in a hurry to get it.

I said she now feels as if she has become more powerful, able to get a response. She agrees, grinning more, telling me she does have friends who would want it, that it was not just a ploy.

She said she had found it easier to get up today but was still late. I wondered if she had a sense of what the lateness was about. She said it was trying to fit too much in. She had been held up by discussing the disposal of rubble with her neighbors.

I said I thought there was a link here with the story about the wood. In that she had turned the situation around. She had something that just didn't work, had a need for something, but it was turned around into something that was the suppliers' problem. They were made to feel the urgency and the need. Maybe when she is late here she is turning it around, so it is me who is to be uncertain and waiting, not her waiting for her time to come.

We maybe learn here something of her early object relationships, in which being in need is felt to be unbearable, might lead to an awful awareness of lack and therefore has to be exported into someone else. One could go further and surmise that in her early experience she felt teased and exploited by the person who has the power to withhold what you need (Kegerreis, 2013, p. 458).”

There can be no doubt reading this extract about the theoretical framework which is being used by the therapist.

### Reflexivity and counter-transference

A good case study contains a high degree of reflexivity, whereby the therapist is able to show his feelings and reactions to the patient's communication in the session and an ability to think about it later with hindsight, by himself or in supervision. This reflexivity needs to show the pattern of the therapist's thinking and how this is related to his school of thought and to his counter-transferential experiences. How has the counter-transference been dealt with in a professional context? One can also consider whether the treatment has been influenced by supervision or discussion with colleagues.

“Recently for a period of a few days I found I was doing bad work. I made mistakes in respect of each one of my patients. The difficulty was in myself and it was partly personal but chiefly associated with a climax that I had reached in my relation to one particular psychotic (research) patient. The difficulty cleared up when I had what is sometimes called a ‘healing’ dream. […] Whatever other interpretations might be made in respect of this dream the result of my having dreamed it and remembered it was that I was able to take up this analysis again and even to heal the harm done to it by my irritability which had its origin in a reactive anxiety of a quality that was appropriate to my contact with a patient with no body (Winnicott, 1949, p. 70).”

### Leaving room for interpretation

A case study is the therapist's perspective on what happened. A case study becomes richer if the author can acknowledge aspects of the story that remain unclear to him. This means that not every bit of reported clinical material should be interpreted and fitted within the framework of the research. There should be some loose ends. Britton and Steiner (1994) refer to the use of interpretations where there is no room for doubt as “soul murder.” A level of uncertainty and confusion make a case study scientifically fruitful (Colombo and Michels, 2007). The writer can include with hindsight what he thinks he has not considered during the treatment and what he thinks could have changed the course for the treatment if he had been aware or included other aspects. This can be seen as an encouragement to continue to be curious and maintain an open research mind.

### Answering the research question, and comparison with other cases

As in any research report, the author has to answer the research question and relate the findings to the existing literature. Of particular interest is the comparison with other similar cases. Through comparing, aggregating, and contrasting case studies, one can discover to what degree and under what conditions, the findings are valid. In other words, the comparison of cases is the start of a process of generalization of knowledge.

“Although based on a single case study, the results of my research appear to concur with the few case studies already in the field. In reviewing the literature on adolescent bereavement, it was the case studies that had particular resonance with my own work, and offered some of the most illuminating accounts of adolescent bereavement. Of special significance was Laufer's (1966) case study that described the narcissistic identifications of ‘Michael’, a patient whose mother had died in adolescence. Both Laufer's research and my own were conducted using the clinical setting as a basis and so are reflective of day-to-day psychotherapy practice (Keenan, 2014, p. 33).”

## Conclusion

As Yinn (2014) has argued for the social sciences, the case study method is the method of choice when one wants to study a phenomenon in context, especially when the boundaries between the phenomenon and the context are fussy. We are convinced that the same is true for case study methodology in the fields of psychoanalysis and psychotherapy. The current focused review has positioned the research method within these fields, and has given a number of guidelines for future case study researchers. The authors are fully aware that giving guidelines is a very tricky business, because while it can channel and stimulate research efforts it can as well-limit creativity and originality in research. Moreover, guidelines for good research change over time and have to be negotiated over and over again in the literature. A similar dilemma is often pondered when it comes to qualitative research (Tracy, 2010). However, our first impetus for providing these guidelines is pedagogical. The three authors of this piece are experienced psychotherapists who also work in academia. A lot of our students are interested in doing case study research with their own patients, but they struggle with the methodology. Our second impetus is to improve the scientific credibility of the case study method. Our guidelines for what to include in the written account of a case study, should contribute to the improvement of the quality of the case study literature. The next step in the field of case study research is to increase the accessibility of case studies for researchers, students and practitioners, and to develop methods for comparing or synthesizing case studies. As we have described above, efforts in that direction are being undertaken within the context of the Single Case Archive.

## Author contributions

JW has written paragraphs 1–4; ER and JW have written paragraph 5 together; SK has contributed to paragraph 5 and revised the whole manuscript.

### Conflict of interest statement

The authors declare that the research was conducted in the absence of any commercial or financial relationships that could be construed as a potential conflict of interest.
